# A PETase enzyme synthesised in the chloroplast of the microalga *Chlamydomonas reinhardtii* is active against post-consumer plastics

**DOI:** 10.1038/s41598-023-37227-5

**Published:** 2023-06-20

**Authors:** Giulia Di Rocco, Henry N. Taunt, Marcello Berto, Harry O. Jackson, Daniele Piccinini, Alan Carletti, Giulia Scurani, Niccolò Braidi, Saul Purton

**Affiliations:** 1 Department of Life Sciences, University of Modena and Reggio Emilia, 41125 Modena, Italy; 2 Algal Research Group, Department of Structural and Molecular Biology, University College London, Gower Street, London, UK; 3 Department of Chemical and Geological Sciences, University of Modena and Reggio Emilia, 41125 Modena, Italy

**Keywords:** Expression systems, Molecular engineering, Protein delivery, Nanoscale biophysics

## Abstract

Polyethylene terephthalate hydrolases (PETases) are a newly discovered and industrially important class of enzymes that catalyze the enzymatic degradation of polyethylene terephatalate (PET), one of the most abundant plastics in the world. The greater enzymatic efficiencies of PETases compared to close relatives from the cutinase and lipase families have resulted in increasing research interest. Despite this, further characterization of PETases is essential, particularly regarding their possible activity against other kinds of plastic. In this study, we exploited for the first time the use of the microalgal chloroplast for more sustainable synthesis of a PETase enzyme. A photosynthetic-restoration strategy was used to generate a marker-free transformant line of the green microalga *Chlamydomonas reinhardtii* in which the PETase from *Ideonella sakaiensis* was constitutively expressed in the chloroplast. Subsequently, the activity of the PETase against both PET and post-consumer plastics was investigated via atomic force microscopy, revealing evidence of degradation of the plastics.

## Introduction

Natural polymers such as lignin, starch, chitin, and cellulose are water-insoluble macromolecules present in the environment, and although such polymers are generally recalcitrant to physical and chemical degradation, nature has evolved enzymes for their breakdown^1^. Plastics are instead synthetic polymers designed specifically for their high resistance to degradation. Plastics are central to modern life and their production has expanded tremendously during the last few decades owing to their versatile properties and low cost. Although an increasing number of microorganisms capable of degrading plastic polymers have been isolated and the enzymes involved metabolically characterized, additional studies are needed to identify novel enzymes and associated degradation pathways for the wide range of different plastics^1–5^. The challenge of understanding and optimizing plastic enzymatic degradation closely emulates that of enzymatic depolymerization of polysaccharides^6–8^. Indeed, strategies that have been used to understand and improve glycoside hydrolases, including the development of quantitative assays for measuring enzyme (or enzyme cocktail) performance, can serve as inspiration for more quantitative metrics for comparing plastic-degrading enzymes and enzyme mixtures^9,10^. Owing to its robust mechanical properties and high post-consumer recycling costs, polyethylene terephthalate (PET) is one of the most abundant plastics in the world. PET accumulates in our environment without significant microbial conversion^2–5^. The constant flux of new PET into the global market has produced enormous amounts of waste with a long biodegradation timescale. This waste contributes to global pollution, especially for marine ecosystems, and poses a threat to human and animal health^11–14^.

In 2016, Yoshida et al. discovered and isolated two new enzymes from *Ideonella sakaiensis,* a bacterium which was able to grow using PET as the main carbon and energy source^15^. These enzymes are a polyethylene terephthalate hydrolase (PETase), which can convert PET into mono(2-hydroxyethyl) terephthalic acid (MHET) and mono(2-hydroxyethyl) terephthalate hydrolase, responsible for the conversion of MHET to terephthalic acid (TPA) and ethylene glycol (EG)^15–18^. Since then, numerous PET hydrolases, belonging to the esterase class (EC 3.1.1., carboxylic ester hydrolases) have been reported and characterized^19–25^. Given that plastic waste is mainly disposed of via incineration or thermal degradation, the possibility of biological degradation of such waste to non-toxic monomers still represents an attractive and greener solution to reduce pollution.

For production of enzymes for research and industrial purposes, the exploitation of the native plastic-degrading microorganism is not always possible and organisms that are easier to culture and engineer for heterologous expression are required^26^. To this end, microalgae represent an attractive biotechnology platform for the synthesis of recombinant proteins^27–29^. The advantages of using microalgae as opposed to traditional heterotrophic platforms of *Escherichia coli*, yeast, or CHO cells are: (i) the low-cost phototrophic cultivation of the alga in sterile, controlled photobioreactors using simple and inexpensive medium^30^ and in this particular case without using any kind of antibiotics for the selection of the strain; (ii) the generally recognized as safe (GRAS) status of a number of algal species, including the chlorophyte *Chlamydomonas reinhardtii*^31^; (iii) the availability of the chloroplast as a unique biosynthetic and storage compartment within the cell that contains its own minimal genetic system^32–34^; and (iv) a growing interest and adoption of enabling synthetic biology principles for creating bespoke cell factories using microalgae^35^. Whilst several recent studies have reported the production of PETase in microalgal species through nuclear engineering^36,37^*,* the use of the chloroplast for expression of foreign genes confers several benefits. These including precise insertion into the chloroplast genome (= “plastome”) via homologous recombination, high-level expression that is not subject to any gene-silencing mechanisms, the possibility of expressing multiple transgenes as operons and the accumulation of the recombinant proteins in a benign compartment where the formation of di-sulfide bonds occurs readily^38^. Here we report the synthesis of PETase from *I. sakaiensis* in the chloroplast of *C. reinhardtii* and demonstrate that the purified enzyme is active against both PET and post-consumer plastic (PCP).

## Results

### Generation of transplastomic *C. reinhardtii* expressing PETase

**Figure 1:**
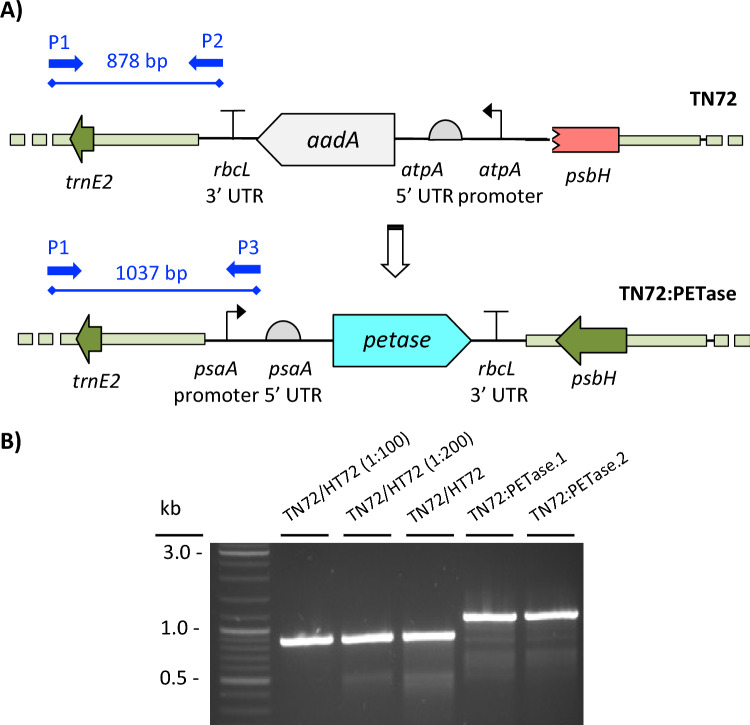
(**A**) Transformation of TN72 with the pSRSapI:PETase plasmid. Phototrophic selection simultaneously restores *psbH* and replaces the *aadA* cassette. with a PETase expression cassette. The coding sequences, promoters and UTR elements of each cassette are illustrated using standard SBOL glyphs^42^. PCR primers used for genotype diagnostics are indicated: primer P1 binds upstream from the insertion site in both genotypes and gives either an 878 bp band in combination with primer P2 (binds to the TN72 plastome) or a 1037 bp band with primer P3 (binds to the transformed plastome). (**B**) PCR results confirming the homoplasmic state of transformants TN72:PETase.1 and TN72:PETase.2. A single band of 878 bp is observed in the TN72 control, whereas a 1037 bp band is detected for the transformant lines. 1:100 and 1:200 dilutions of the TN72 DNA validate the sensitivity of the assay for detecting even a single copy of the parental DNA remaining in the transformant chloroplast.

A synthetic gene encoding the mature form of the *I. sakaiensis* PETase was codon optimized for expression in the chloroplast and cloned into the pSRSapI destination vector, to generate plasmid pSRSapI:PETase (Supplementary Fig. S1). The bacterial PETase enzyme contains two disulphide bonds that are formed following secretion into the *I. sakaiensis* periplasm^39^. We therefore decided to retain the sequence for the *N*-terminal Sec-type signal peptide within the transgene design so that the enzyme would be similarly targeted into the thylakoid lumen. Previous work has shown that bacterial signal peptides can direct recombinant proteins into the thylakoid lumen, and that disulphide bond formation occurs more readily in this chloroplast compartment^40^. This was used to transform *C. reinhardtii* strain TN72 with the *PETase* gene integrated into the plastome at a neutral locus between *psbH* and *trnE2*. Integration of the transforming DNA into TN72 also restores a wild-type copy of *psbH*, which is an essential photosynthesis gene^41^. Selection is therefore based on restoration of photosynthesis allowing the generation of transformants lacking any antibiotic-based selectable marker, with the only foreign DNA introduced into the plastome being the PETase coding sequence (Fig. 1A).

As the *C. reinhardtii* plastome is polyploid with ~ 40 copies per cell under phototrophic conditions^43^, transformant lines were restreaked several times to single colonies under selective conditions to drive the lines to homoplasmy where all plastome copies have the transgenic DNA. Two such lines (TN72:PETase.1 and TN72:PETase.2, hereafter) were checked for homoplasmy using a 3-primer PCR screen (Fig. 1A). This showed a single band at 1037 bp in the TN72:PETase.1 and TN72:PETase.2 PCRs and a single band at 878 bp in the parental TN72 control PCRs, confirming correct integration of the PETase cassette and homoplasmy (Fig. 1B). Furthermore, it was found that selection based on restoration of phototrophy established homoplasmy much more readily than antibiotic-based selection. This is probably due to the strong selective pressure of restoring the photosynthetic phenotype and to the reduced copy number of cells grown on minimal medium rather than the acetate-containing medium normally used for antibiotic-based selection^41^. Finally, PCR amplification and sequencing of the PETase cassette from both transformant lines confirmed than neither had acquired any mutations during plastome integration.

### PETase is expressed in the chloroplast

The TN72:PETase.1 cell line was characterized further in order to assess the level of recombinant protein produced and to determine whether the protein is correctly folded and functional. An evaluation of the PETase level was conducted by SDS-PAGE analysis of cell extracts with proteins bands in the 27 kDa region cut from the gel, trypsin-digested and subjected to tandem mass (MS/MS) spectrometry. As shown in Supplementary Fig. S2 protein sequence coverage for PETase of 27% derived from the cut band of the gel in Supplementary Fig. S2 confirmed unambiguously the presence of PETase in the strains. Other bands from constitutive proteins were analysed revealing the presence of malate dehydrogenase of 30 kDa (Supplementary Fig. S3) and cytochrome *c* at 10 kDa (Supplementary Fig. S4).

### Purification of recombinant PETase from transgenic *C. reinhardtii*

**Figure 2:**
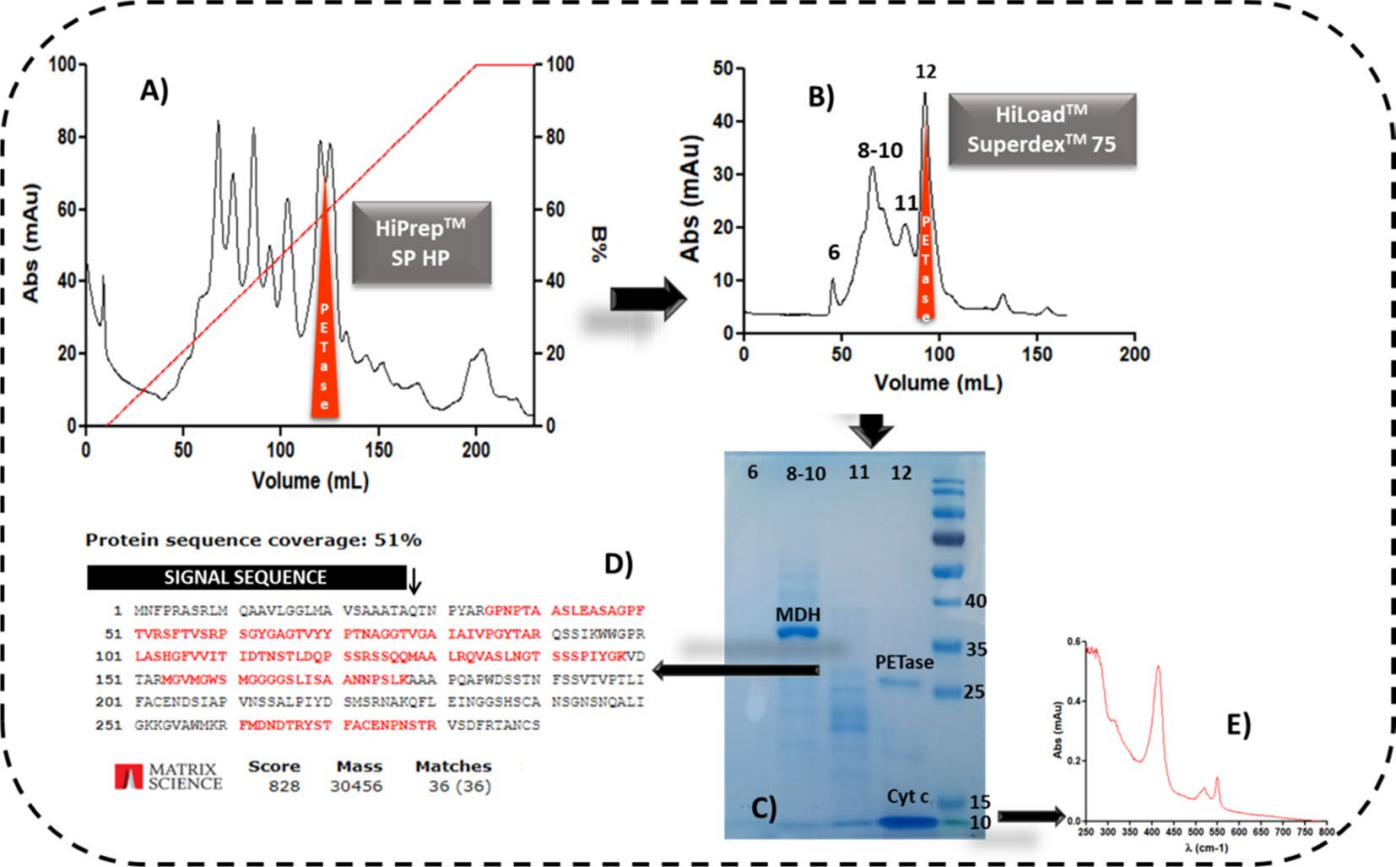
PETase purification pipeline. (**A**) HiPrep™ SP HP 16/10 chromatography (**B**) Size exclusion chromatography (SEC-Superdex 75) was used to produce the final PETase purified product. Recombinant PETase is eluted in fraction 12 (highlighted in red), with significant enrichment of malate dehydrogenase (MDH) seen in fraction 8–10. (**C**) SDS-PAGE for the fractions eluted during HiLoad™ Superdex™ 75 chromatography; the bands of MDH (malate dehydrogenase), PETase and cytochrome *c* were cut from the gel and the digested peptides extracted for MS analysis (**D**) MS/MS result for the PETase protein sequence coverage. (**E**) UV–Vis spectrum with the peaks typical of a cytochrome *c*.

PETase has the classic α/β hydrolases fold, but despite the sequence similarity to cutinases and lipases, the highly polarized surface charge of PETase creates a dipole that gives the enzyme an isoelectric point of 9.6^17^ that allows a first step of purification by cationic exchange. The protein was successfully recovered using an optimized two-step chromatography approach as described in the methods and shown in Fig. 2A,B. Elution of PETase from the first column occurred at a concentration between 0.1 and 0.15 M of NaCl. Several other peaks were observed on the chromatogram during this phase indicating the presence of several endogenous protein contaminants. Fractions highlighted in red in Fig. 2A were loaded on the size exclusion chromatography (SEC) column, and a group of low-concentration proteins eluted first (peak 6 in Fig. 2B); followed by the majority of the proteins as three separate peaks labelled 8–10 (due to the presence of two small shoulders), 11 and 12 (Fig. 2B). All fractions were analyzed by SDS-PAGE (Fig. 2C). The gel shows that fraction 8–10 contained a major protein with a molecular weight greater than 35 kDa, that was analysed by MS/MS revealing the presence of malate dehydrogenase (Supplementary Fig. S3). Sample 12 revealed a band with a molecular weight of approximately 27 kDa, consistent with that of mature PETase. The band was analysed via MS/MS spectrometry confirming its identity as PETase with a 51% sequence coverage (Fig. 2D). Sample 12 also contained a mitochondrial cytochrome *c* that was identified by MS/MS spectrometry and UV–visible spectroscopy (Supplementary Fig. S4 and Fig. 2E). Since this cytochrome has a pI = 9.39, it eluted with PETase during the cationic exchange chromatography. Moreover the difference in molecular weights between PETase (27 kDa) and cytochrome *c* (12 kDa) was not sufficiently different for the two proteins to be efficiently separated by size exclusion chromatography.

### The recombinant PETase is active

**Figure 3:**
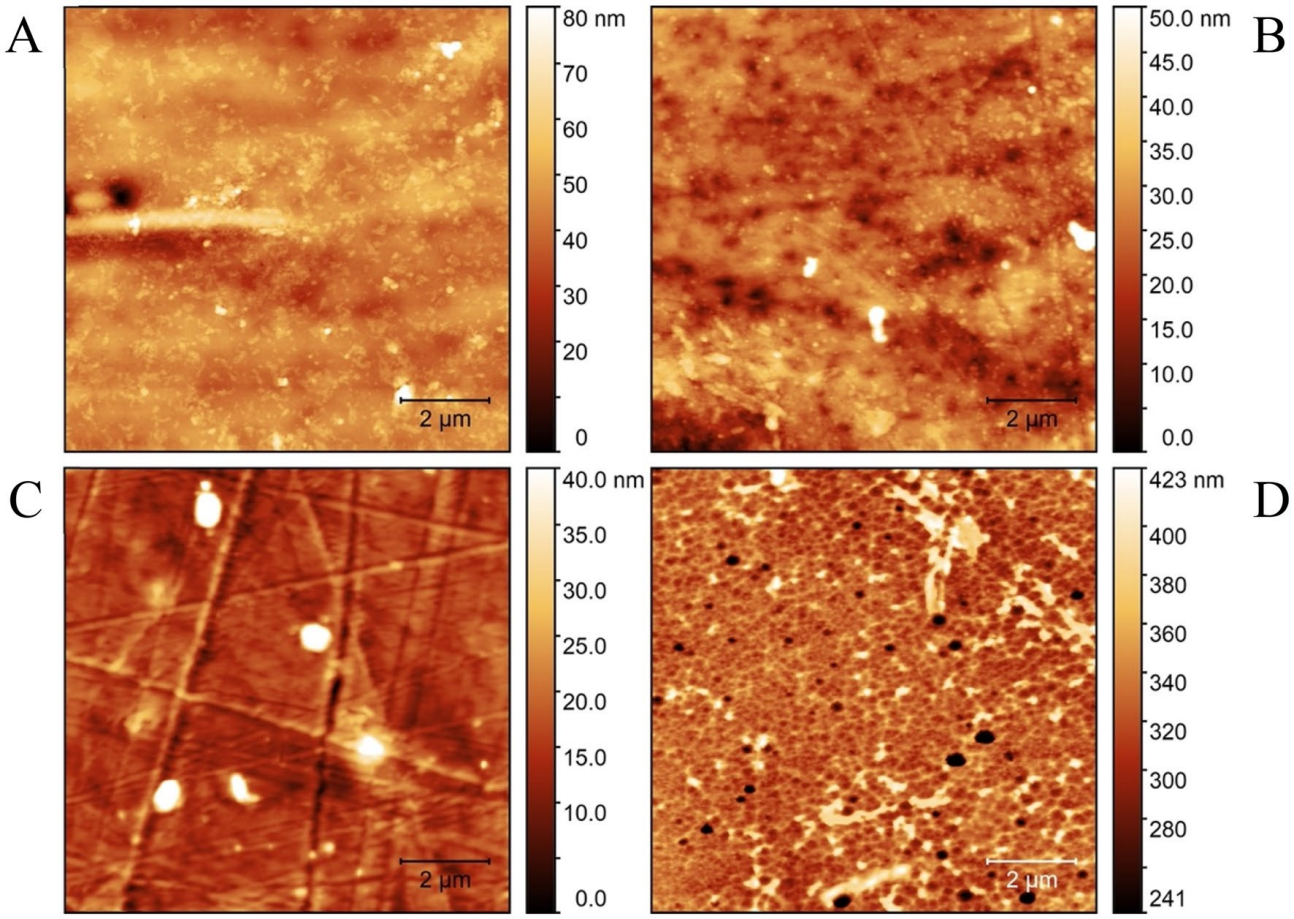
AFM topographical images of PET film and PCP samples following incubation with either recombinant PETase or a control solution. (**A**) PET treated with a control solution, and (**B**) treated with recombinant PETase. (**C**) PCP treated with a control solution, and (**D**) treated with recombinant PETase. All images are 10 µm × 10 µm.

**Figure 4:**
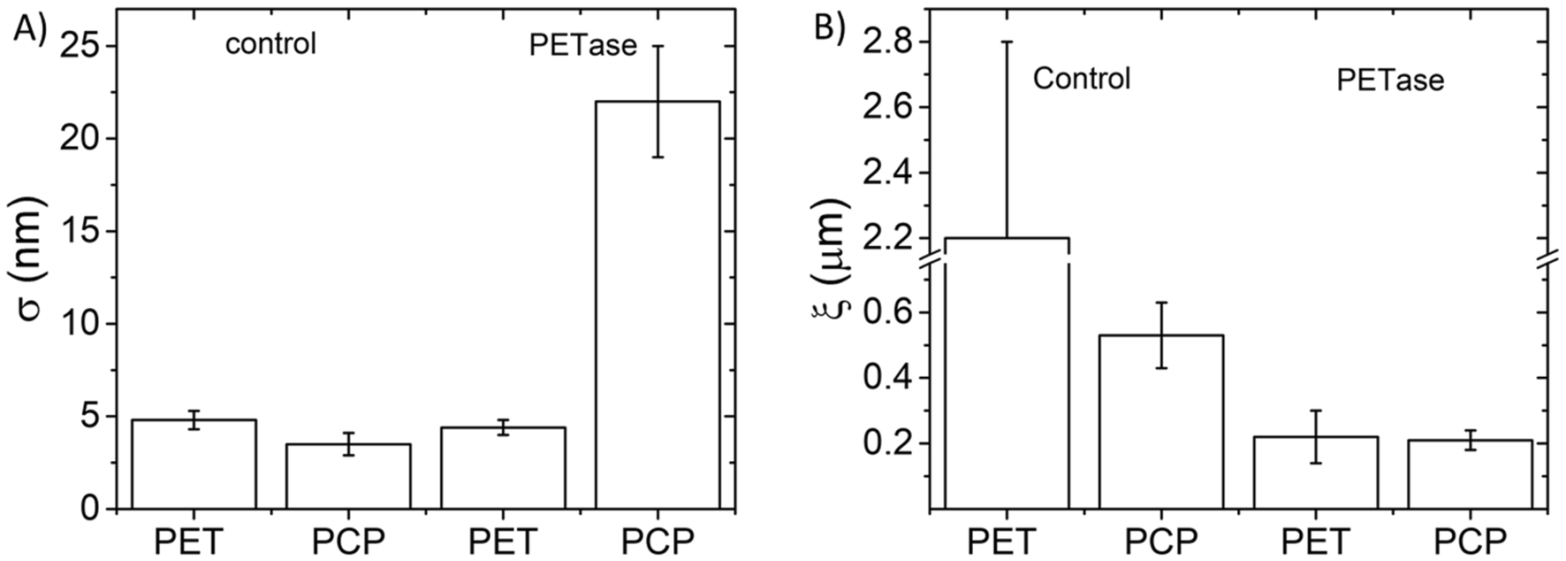
Morphological parameters extracted from AFM images. (**A**) Roughness *σ*_*rms*_ and (**B**) correlation length *ξ* variation between PET and PCP sample after immersion in control and PETase-containing solutions. Standard deviations from 3*n* technical replicate images of one substrate sample are reported as errors.

To investigate the activity of the algal-expressed PETase, PET pure film and post-consumer plastic (PCP) substrates were incubated with the enzyme and control solutions then assessed by semi-contact atomic force microscopy (AFM) imaging in air (Fig. 3). It is clear that the recombinant PETase can modify the morphology of both PET and PCP samples. Specifically, in samples incubated with the enzyme, the formation of holes can be observed by the presence of darker spots, while no holes are visible in the control samples (i.e. the same experimental procedures without the enzyme). To quantitatively evaluate the morphological changes on the surfaces due to PETase activity, the average surface roughness of the samples *σ*_*rms*_ and the lateral correlation length *ξ* were calculated. These variables correspond to a measure of the texture of the surface, and the distance range over which points in one region of space are correlated with those in another region, respectively^44^ (Fig. 3). PETase activity resulted in a decrease of *ξ* in both materials: PET film samples (Fig. 3A,B) displayed a decrease by an order of magnitude, from 2.2 ± 0.6 µm (control sample) to 0.22 ± 0.08 µm (when incubated with enzyme), while, for PCP samples (Fig. 3C,D), a reduction of 60% was observed, from 0.53 ± 0.1 µm (control sample) to 0.21 ± 0.03 µm (when incubated with enzyme). The average values of *ξ* were obtained from three images of each sample and interestingly, after incubation with the enzyme, they were similar (≅ 0.2 µm) for both materials (Fig. 4B). Since there were no other sources of degradation in sample reactions we could ascribe those values exclusively to the PETase activity, and, in particular, the presence, the dimension and the density of the holes on the surfaces confirmed the functionality of the enzyme. Conversely, the surface roughness variation (Δ*σ*_*rms*_) significantly increased for PCP samples (from 4.5 ± 0.6 nm to 22 ± 3 nm with Δ*σ*_*rmsPCP*_ =  + 17.5 nm), while it does not change for PET film samples (from 4.8 ± 0.5 nm to 4.4 ± 0.4 nm with Δ*σ*_*rmsPET*_ = − 0.4 nm) (Fig. 4A,B). This may suggest that the chemical and physical stress suffered by the plastic samples during the incubation could affect more one material than the other. A possible explanation is that the technical-grade PET foil is more resilient to stress than postconsumer plastic foil (in fact Δ*σ*_*rmsPCP*_ < Δ*σ*_*rmsPET*_), but, in any case both materials were affected by the enzyme activity.

**Figure 5:**
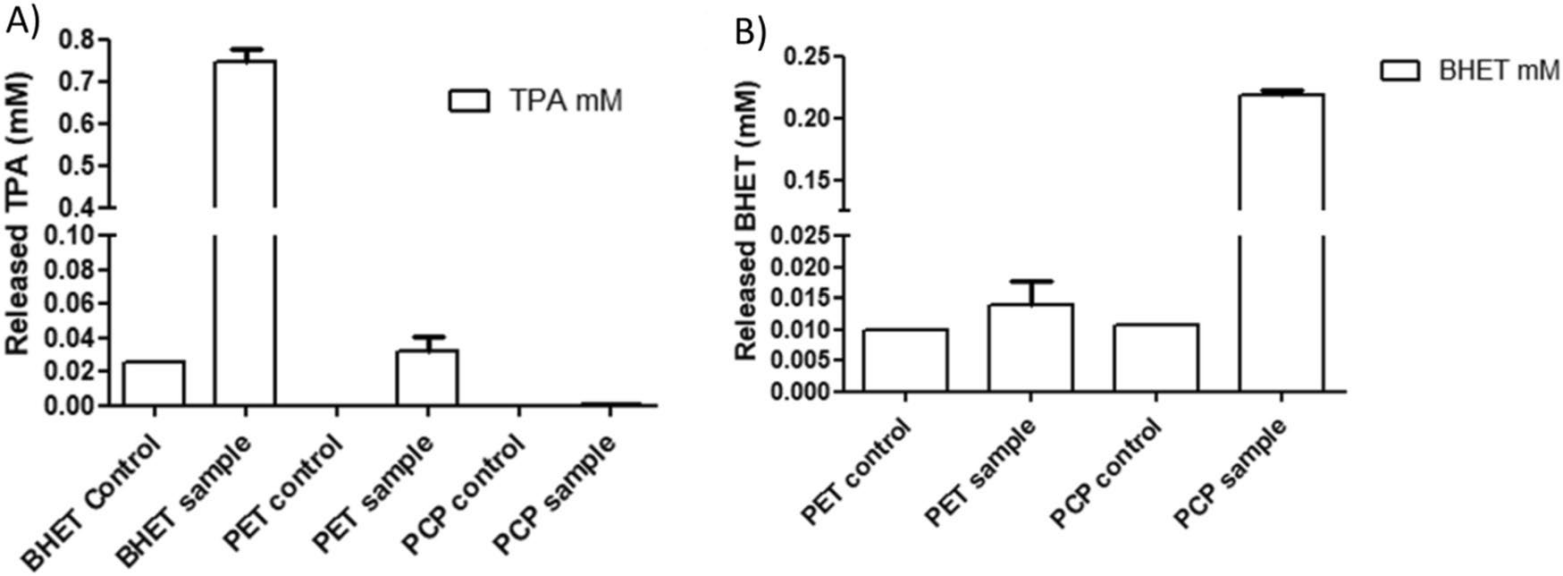
PETase activity in terms of concentration of (**A**) TPA and (**B**) BHET released. The experiments were conducted in phosphate buffer (pH 7) at 30 °C for all the experiments All measurements were conducted in duplicate (n = 2).

To corroborate the analysis of the enzyme activity, the PET film was transformed into powder and PETase was incubated for 48 h in the presence of 2 mg of PET and BHET powder using the same experimental conditions tested before. Simultaneously, the post-consumer plastic (PCP) was similarly tested. The results are summarized in Fig. 5 and the calibration curves for TPA and BHET standards are in supplementary material (Supplementary Figs. S7 and S8a,b) together with two chromatograms from the reaction supernatants (Supplementary Fig. S8c,d). As expected, PETase was active on all the polymer samples. PET powder digestion yielded 0.02 mM of TPA which is line with the data in the literature for the WT protein^45^. The BHET powder was not completely digested thus it was not possible to obtain the MHET data for standard values acquisition; nevertheless it produced 0.8 mM of monomer when in contact with the enzyme (Fig. 5A). For the PCP sample at a reaction time of 48 h it was not possible to detect any significantly amount of TPA since the reaction was conducted at low temperature (30 °C) and for a relatively short period but the main product released by the PCP sample was BHET at 0.20 mM (Fig. 5B).

## Discussion

PETase is a recently discovered hydrolase enzyme acting on PET and many advances have already been made to increase PETase catalytic activity and its ability to work at high temperatures^15,19–22,24,25,45^. With a view to its potential significance in an industrial context and the need to discover new PETases from various organisms in this work we describe a platform for producing such enzymes in the chloroplast of C. *reinhardtii.* Here we obtained ~ 5 mg of recombinant *I. sakaiensis* PETase (although, not completely pure) from 40 g of wet biomass (yield of purified mixture ≅ 0.012%)*.* While the recombinant protein yield is lower than that of bacterial systems^46^, the activity of the algal-expressed enzyme in terms of concentration of released TPA is in line with the published data for the wt protein^45,47^. Moreover, microalgae offer several potential advantages, such as reduced scale-up costs and use of the algal biomass for extraction of useful byproducts; carotenoids, pigments, proteins, and vitamins that can be used for the production of nutraceuticals, animal feed additives, cosmetics, or for energy production^29,48–51^. Expression in the chloroplasts has the advantage of precision targeting of transgenic DNA into a selected site in the plastome via homologous recombination, stable and high-level expression of transgenes in the absence of selection, and the ability to compartmentalize and target potentially toxic recombinant proteins within the chloroplast. Moreover, the use of photosynthetic restoration in the transformation of strain TN72 avoids the use of selection markers based on antibiotic-resistance genes and circumvents concerns over escape of such genes via lateral gene transfer. Finally, the pilot scale PETase production carried out here (10 L culture medium), allowed for the first time the expression of a sufficient amount of recombinant protein for purification, and this will facilitate further characterization of the enzyme kinetics for different PETase variants with improved PET activity^19^. Interestingly, the purification of the recombinant PETase by means of cationic exchange chromatography resulted in the presence of a cytochrome *c* with a pI similar to that of the PETase (9.39 and 9.65, respectively) in the final fraction, implying that the two proteins stayed together during all the purification steps. Considering this result, it is worth considering if the cytochrome *c* could affect the activity of PETase enzyme in the reaction mixture. Cytochrome *c* is a well characterized and ubiquitous protein that can interact with various molecules (i.e. phospholipids through electrostatic and hydrophobic bonds)^52–55^, and in some cases (e.g. a M80A mutant) it can exert a catalytic activity^56–58^, but primarily cytochrome *c* is an electron transfer protein working only in the presence of electron donors that, in this specific case, were not added to the reaction mixture. Therefore, we can exclude the possibility that the cytochrome *c* contributed to the PETase enzymatic reaction. Moreover, the reaction yields measured are in accordance with those of the pure enzyme.

The aim of this work was the production of an active PETase in the microalgal chloroplast. This first successful demonstration of transgenic strain of C. *reinhardtii* capable of synthesizing the *I. sakaiensis* PETase into an active form, paves the way for future studies focused on production of improved PETase variants and other plastics-degrading enzymes in this sustainable platform.

## Methods

### Materials

*Chlamydomonas reinhardtii* strain TN72 (CC-5168, Chlamydomonas Resource Center: www.chlamycollection.org) was used to generate the transgenic lines in this study as described previously^41,59^. All lines were maintained on tris-acetate-phosphate (TAP) medium supplemented with 1.5% agar unless otherwise stated. Liquid cultures were cultivated in TAP medium at 25 °C, with agitation at 125 rpm. All lines were illuminated with continuous white light at 50 μE/m^2^/s, with the light sensitive TN72 parental line protected with a white paper cover to reduce light intensity by approximately an order of magnitude.

### Cloning procedures

DNA manipulations were performed following standard protocols^60^, including transformation of chemically competent *E. coli* DH5α. Oligonucleotide primers were purchased from Eurofins Genomics (Ebersberg, Germany) while restriction enzymes and T4 DNA ligase for cloning procedures are purchased from NEB (Ipswich, MA, USA) and Thermo Scientific (Waltham, MA, USA). The coding sequence for *I. sakaiensis* PETase (PETase Uniprot: A0A0K8P6T7; Gene: ISF6_4831; EC:3.1.1.101), together with a Strep-tag at the N terminus, was codon-optimized for the *C. reinhardtii* chloroplast using the Codon Usage Database (www.kazusa.or.jp/codon) with SapI and SphI sites added for cloning into the pSRSapI transformation vector (Supplementary Fig. S1)^61^. The DNA was synthesized by IDT (Integrated DNA Technologies, Inc., Coralville, Iowa-USA), and was cloned into the transformation vector by standard molecular techniques^60^.

### Transformation of the *C. reinhardtii* chloroplast

*Chlamydomonas reinhardtii* was transformed by the glass bead method as described previously^41,62^. 400 mL transformation cultures were grown to early log phase (1–2 × 10^6^ cells/mL) and harvested by centrifugation at 3000×*g*. Cells were resuspended in sterile high salt minimal (HSM) medium to a density of 2 × 10^8^ cells/mL, and 300 μL added to sterilized 5-mL tests tube containing 300 mg sterile 400–625 μm diameter glass beads and 10 μg plasmid DNA. Tubes were agitated by vortex at maximum speed for 15 s, then mixed with 4 mL molten HSM supplemented with 0.5% agar at 42 °C and immediately spread on HSM plates supplemented with 1.5% agar and 100 μg/mL ampicillin. Plates were sealed with parafilm and incubated at 25 °C in dim light (~ 2 μE/m^2^/s) overnight then transferred to moderate light (~ 50 μE/m^2^/s)^32,41^. Transformant colonies were picked after 3–4 weeks and restreaked to single colonies on selective media until homoplasmy was achieved, typically after 2–3 restreaks.

### Genotyping of transformant lines

Lines were assessed by PCR analysis using a three-primer strategy as described previously^41^. In each case a forward flanking primer was designed outside of the left homology arm used for transformation, with reverse primers designed within the parental and transformed cassettes respectively. Details of primers used are given in the supplementary data (Supplementary Table S1). Insertion of the target PETase CDS was further confirmed by PCR amplification of the expression cassette followed by Sanger sequencing of the PCR product. Total genomic DNA is extracted from a small amount of cells using the Chelex 100 method^63^ and PCR amplification is carried out with Phusion DNA polymerase (Thermo Scientific) according to the manufacturer’s instructions. In this strategy (see Fig. 1A), a plastome specific primer (P1) is used in conjunction with two other primers in a three primer reaction. A second primer (P2) binds to a terminator element (*T*_*rbcL*_) in the parental TN72 strain, in which the endogenous *psbH* gene (essential for photosynthesis) is disrupted with an aminoglycoside 3’-adenylyltransferase (aadA) expression cassette, to generate a band of 878 bp. The third primer (P3) binds to a promoter element (*P*_*psaA*_) in the transformed genotype, containing the PETase expression cassette and the restored of *psbH* to give a band of 1037 bp. Dilution control PCR (1:100 and 1:200) were performed with TN72 DNA, to ensure that the PCR reaction was sufficiently sensitive to detect the parental DNA in the TN72:PETase PCR if the parental DNA were present at low/single copy numbers.

### Scale up cultivation, cell breakage, and preparation of soluble protein extract

*Chlamydomonas reinhardtii* expressing PETase cells line was inoculated in 20 mL of liquid TAP medium and grown at, 25 °C, 100 rpm and intensity light of 50 μE m^−2^ s^−1^ for 4 days. Cell growth was monitored over time using a Bürker chamber and the rate of increase was obtained. Cells were let grown until a concentration of 4 × 10^6^ cells/mL was reached and then an inoculum of 2 × 10^5^ cells/mL was scaled twice, each with 1:10 dilutions. A further scale up was conducted using a 10 L homemade bioreactor. Cells were harvested by centrifugation at 3000*g*, 4 °C for 15 min and disrupted by 20 sonication cycles of 1 min each. In order to remove cell debris and hydrophobic proteins, a precipitation step in presence of (NH_4_)_2_SO_4_ 1 M was then followed by dialysis *vs* 25 mM phosphate buffer pH 7 and finally loaded into the column.

### Protein purification

PETase was purified exploiting the calculated pI of 9.6 and the molecular weight of 27,559 Da, using a two-step reliable purification protocol with a cationic exchange HiPrep™ SP HP 16/10 (Cytiva) chromatography. The eluted fractions containing PETase were collected and concentrated by Amicon^®^ membrane ultrafiltration spin columns with a 5000 Da cutoff to a < 2 mL volume, and loaded on a size exclusion SEC separation using a HiLoad™ Superdex™ 75 (GE Healthcare) column. PETase was eluted from the HiPrep™ SP HP 16/10 column across a 25 mM sodium posphate buffer pH 7/25 mM sodium posphate buffer pH 7, 0.5 M NaCl gradient. Fractions containing the protein were pooled, concentrated, and loaded onto a SEC HiLoad™ Superdex™ 75 column and eluted isocratically in the presence of 200 mM phosphate buffer pH 7; 0.15 M NaCl. The eluted fractions from the two chromatographic steps were assessed by SDS-PAGE and western blotting.

### Tandem mass spectrometry

For UHPLC–HRMS analysis, dry extracted peptides were resuspended in 50 μL of a mixture of water:acetonitrile:formic acid 95:3:2, sonicated for 10 min at room temperature and centrifuged at 12,100*g* for 10 min. A Thermo Scientific Dionex Ultimate 3000 195 UHPLC coupled to a Thermo high-resolution Q Exactive mass spectrometer (Thermo Scientific, Bremen, Germany) was used for the analyses. Centroided MS and MS2 spectra were recorded from 200 to 2000 *m/z* in Full MS/dd-MS^2^ (TOP2) mode. Precursor dynamic exclusion (6 s) and apex triggering (1–5 s) were set; peptide-like isotope pattern ions were preferred. The mass spectrometer was calibrated before the start of the analyses; an initial segment (0.1–0.7 min) with a lock mass (391.28429) was included in the MS 204 method. For protein identification, raw data, converted into mascot generic format using MsConvert (v. 3.0.10730, ProteoWizard tools; 25), were searched against Swiss-Prot for peptide sequences and an in-house database. Trypsin was selected as the proteolytic enzyme; oxidized methionine (M) was set as variable modifications while carbamidomethylation of cysteine (C) was set as fixed modifications in the search parameters. One missed cleavage was allowed. Mass tolerances were set at 10 ppm for the precursor ions (peak detection mismatch #13C = 1) and 0.5 Da for all the samples. An automatic decoy database search was used to estimate the false discovery rate; probability threshold was trimmed to get a FDR < 1%.

### Activity assays

Purified recombinant PETase was tested against 0.25 mm thick polyethylene terephthalate (PET) film (Goodfellow Cambridge Ltd) and post-consumer packaging (PCP). 200 μL digestion reactions were set up with 6 × 4 mm pieces of clean substrate, 10 μL of purified protein fraction or a control solution in Phosphate buffer 25 mM pH 7. Reaction tubes were incubated at 30 °C shaking (250 rpm) for 96 h. Solid substrate was then removed and rinsed in subsequent steps with 1% SDS. Protein activity was assessed by analyzing the plastic pieces after incubation with the enzyme by atomic force microscopy (AFM). Morphological characterization of PET and PCP samples was performed using an NT-MDT SMENA Solver platform (Moscow, Russia); the analysis was performed in semi-contact mode and the images analyzed using Gwyddion 2.61 freeware (http://gwyddion.net). PET powder was obtained as following; polyethylene terephthalate (16.67 mg of PET film) and 2 mL of p-cresol were placed in a round-bottom flask equipped with a magnetic stirrer. The flask was immersed in a preheated (70 °C) oil bath and the mixture was stirred at 500 rpm for 1 h. After PET solubilization, the solution was dropwise added to 100 mL of ethanol under vigorous stirring. The precipitate was separated by filtration, then washed several times with distilled water, and finally dried in an oven (70 °C).

### HPLC analysis

PET hydrolysis products were desiccated and resuspended in 50 μL of DMSO and then loaded into the HPLC System. The separation occurred using an Agilent 1260 Infinity II HPLC System equipped with a G7114A 1260 VWD UV–Vis detector and an Agilent InfinityLab Poroshell 120 C18 column (Thermo Scientific™). An isocratic mobile phase consisting of 80% of (10%formic acid (v/v)) solvent (A) and 20% of acetonitrile (solvent B) was used at a flow rate of 1 mL/min during a 15 min gradient which gradually shifted the mobile phase composition to 50% of acetonitrile. The concentrations of hydrolyzed products (BHET, MHET, and TPA) were detected at 254 nm and calculated from the areas of the adsorption peaks using calibration curves established from TPA and BHET standard solutions. The retention times of TPA, and BHET were about 1.65, and 2.4 min, respectively (Supplementary Fig. S9a,b). The MHET obtained by hydrolyzing BHET standard solution using PETase has a retention time of 2.056 (Supplementary Fig. S9c,d).

## Supplementary Information

Supplementary Figures.

## Data Availability

The datasets used or analysed are available on https://zenodo.org/badge/DOI/10.5281/zenodo.7956450.svg. https://doi.org/10.5281/zenodo.7740875.

## References

[1] Chen CC, Dai L, Ma L, Guo RT (2020). Enzymatic degradation of plant biomass and synthetic polymers. Nat. Rev. Chem..

[2] Shah AA, Hasan F, Hameed A, Ahmed S (2008). Biological degradation of plastics: A comprehensive review. Biotechnol. Adv..

[3] Pathak, V. M. & Navneet. Review on the current status of polymer degradation: A microbial approach. *Bioresour. Bioprocess.* **4**, (2017).

[4] Kaushal J, Khatri M, Arya SK (2021). Recent insight into enzymatic degradation of plastics prevalent in the environment: A mini-review. Clean. Eng. Technol..

[5] Tokiwa Y, Calabia BP, Ugwu CU, Aiba S (2009). Biodegradability of plastics. Int. J. Mol. Sci..

[6] Hemsworth GR, Henrissat B, Davies GJ, Walton PH (2014). Discovery and characterization of a new family of lytic polysaccharide mono-oxygenases. Nat. Chem. Biol..

[7] Müller G, Várnai A, Johansen KS, Eijsink VGH, Horn SJ (2015). Harnessing the potential of LPMO-containing cellulase cocktails poses new demands on processing conditions. Biotechnol. Biofuels.

[8] Vaaje-kolstad G (2010). An oxidative enzyme boosting the. Science.

[9] Serra I (2022). Activity and substrate specificity of lytic polysaccharide monooxygenases: An ATR FTIR-based sensitive assay tested on a novel species from *Pseudomonas putida*. Protein Sci..

[10] Breslmayr E (2018). A fast and sensitive activity assay for lytic polysaccharide monooxygenase. Biotechnol. Biofuels.

[11] Cózar A (2014). Plastic debris in the open ocean. Proc. Natl. Acad. Sci. U. S. A..

[12] Worm B, Lotze HK, Jubinville I, Wilcox C, Jambeck J (2017). Plastic as a persistent marine pollutant. Annu. Rev. Environ. Resour..

[13] Gregory MR (2009). Environmental implications of plastic debris in marine settings-entanglement, ingestion, smothering, hangers-on, hitch-hiking and alien invasions. Philos. Trans. R. Soc. B. Biol. Sci..

[14] Halden RU (2010). Plastics and health risks. Annu. Rev. Public Health.

[15] Yang Y, Yang J, Jiang L (2016). Comment on "a bacterium that degrades and assimilates poly(ethylene terephthalate) ". Science (80-)..

[16] Austin HP (2018). Characterization and engineering of a plastic-degrading aromatic polyesterase. Proc. Natl. Acad. Sci. U. S. A..

[17] Knott BC (2020). Characterization and engineering of a two-enzyme system for plastics depolymerization. Proc. Natl. Acad. Sci. U. S. A..

[18] Meyer-Cifuentes IE, Öztürk B (2021). Mle046 is a marine mesophilic MHETase-like enzyme. Front. Microbiol..

[19] Tournier V (2023). Enzymes’ power for plastics degradation. Chem. Rev..

[20] Tournier V (2020). An engineered PET depolymerase to break down and recycle plastic bottles. Nature.

[21] Buchholz PCF (2022). Plastics degradation by hydrolytic enzymes: The plastics-active enzymes database—PAZy. Proteins Struct. Funct. Bioinform..

[22] Puspitasari N, Tsai SL, Lee CK (2021). Class I hydrophobins pretreatment stimulates PETase for monomers recycling of waste PETs. Int. J. Biol. Macromol..

[23] Ronkvist ÅM, Xie W, Lu W, Gross RA (2009). Cutinase-catalyzed hydrolysis of poly(ethylene terephthalate). Macromolecules.

[24] Pirillo V, Orlando M, Tessaro D, Pollegioni L, Molla G (2022). An efficient protein evolution workflow for the improvement of bacterial PET hydrolyzing enzymes. Int. J. Mol. Sci..

[25] Wei R (2022). Mechanism-based design of efficient PET hydrolases. ACS Catal..

[26] Gaber Y (2020). Heterologous expression of lytic polysaccharide monooxygenases (LPMOs). Biotechnol. Adv..

[27] Gong Y, Hu H, Gao Y, Xu X, Gao H (2011). Microalgae as platforms for production of recombinant proteins and valuable compounds: Progress and prospects. J. Ind. Microbiol. Biotechnol..

[28] Rasala BA, Mayfield SP (2015). Photosynthetic biomanufacturing in green algae; Production of recombinant proteins for industrial, nutritional, and medical uses. Photosynth. Res..

[29] Dyo YM, Purton S (2018). The algal chloroplast as a synthetic biology platform for production of therapeutic proteins. Microbiol. (U.K.).

[30] Changko S, Rajakumar PD, Young REB, Purton S (2020). The phosphite oxidoreductase gene, ptxD as a bio-contained chloroplast marker and crop-protection tool for algal biotechnology using Chlamydomonas. Appl. Microbiol. Biotechnol..

[31] Murbach TS (2018). A toxicological evaluation of *Chlamydomonas reinhardtii*, a Green Algae. Int. J. Toxicol..

[32] Economou, C., Wannathong, T., Szaub, J., Purton, S. *Chloroplast Biotechnol*. (2014). 10.1007/978-1-62703-995-6_27.

[33] Taunt HN, Stoffels L, Purton S (2018). Green biologics: The algal chloroplast as a platform for making biopharmaceuticals. Bioengineered.

[34] Bateman JM, Purton S (2000). Tools for chloroplast transformation in Chlamydomonas: Expression vectors and a new dominant selectable marker. Mol. Gen. Genet..

[35] Jackson HO, Taunt HN, Mordaka PM, Smith AG, Purton S (2021). The algal chloroplast as a testbed for synthetic biology designs aimed at radically rewiring plant metabolism. Front. Plant Sci..

[36] Moog D (2019). Using a marine microalga as a chassis for polyethylene terephthalate (PET) degradation. Microb. Cell Fact..

[37] Kim JW (2020). Functional expression of polyethylene terephthalate-degrading enzyme (PETase) in green microalgae. Microb. Cell Fact..

[38] Tran M, Zhou B, Pettersson PL, Gonzalez MJ, Mayfield SP (2009). Synthesis and assembly of a full-length human monoclonal antibody in algal chloroplasts. Biotechnol. Bioeng..

[39] Seo H (2019). Production of extracellular PETase from Ideonella sakaiensis using sec-dependent signal peptides in *E. coli*. Biochem. Biophys. Res. Commun..

[40] Bally J (2008). Both the stroma and thylakoid lumen of tobacco chloroplasts are competent for the formation of disulphide bonds in recombinant proteins. Plant Biotechnol. J..

[41] Wannathong T, Waterhouse JC, Young REB, Economou CK, Purton S (2016). New tools for chloroplast genetic engineering allow the synthesis of human growth hormone in the green alga *Chlamydomonas reinhardtii*. Appl. Microbiol. Biotechnol..

[42] McLaughlin JA (2020). The synthetic biology open language (SBOL) Version 3: Simplified data exchange for bioengineering. Front. Bioeng. Biotechnol..

[43] Lau KW, Ren JWM (2000). Redox modulation of chloroplast DNA replication in *Chlamydomonas reinhardtii*. Antioxid Redox Signal.

[44] Di Lauro M (2017). Liquid-gated organic electronic devices based on high-performance solution-processed molecular semiconductor. Adv. Electron. Mater..

[45] Lu H (2022). Machine learning-aided engineering of hydrolases for PET depolymerization. Nature.

[46] Rosano GL, Morales ES, Ceccarelli EA (2019). New tools for recombinant protein production in *Escherichia coli*: A 5-year update. Protein Sci..

[47] Dai L (2021). Catalytically inactive lytic polysaccharide monooxygenase PcAA14A enhances the enzyme-mediated hydrolysis of polyethylene terephthalate. Int. J. Biol. Macromol..

[48] Chisti Y (2007). Biodiesel from microalgae. Biotechnol. Adv..

[49] Spolaore P, Joannis-Cassan C, Duran E, Isambert A (2006). Commercial applications of microalgae. J. Biosci. Bioeng..

[50] Deng Y (2021). Microalgae for nutrient recycling from food waste to aquaculture as feed substitute: A promising pathway to eco-friendly development. J. Chem. Technol. Biotechnol..

[51] Fabris, M. *et al.* Emerging technologies in algal biotechnology: toward the establishment of a sustainable, algae-based bioeconomy. *Front. Plant Sci.* **11**, (2020). 10.3389/fpls.2020.00279 PMC7090149 32256509

[52] Parray ZA (2021). Interaction of polyethylene glycol with cytochrome c investigated via in vitro and in silico approaches. Sci. Rep..

[53] Krasnikov BF (2011). Synthetic and natural polyanions induce cytochrome c release from mitochondria in vitro and in situ. Am. J. Physiol. Cell Physiol..

[54] Ranieri A (2015). Immobilized cytochrome c bound to cardiolipin exhibits peculiar oxidation state-dependent axial heme ligation and catalytically reduces dioxygen. J. Biol. Inorg. Chem..

[55] Di Rocco G (2021). The enthalpic and entropic terms of the reduction potential of metalloproteins: Determinants and interplay. Coord. Chem. Rev..

[56] Ranieri A (2019). Electrocatalytic properties of immobilized heme proteins: Basic principles and applications. ChemElectroChem.

[57] Lancellotti L (2020). Adsorbing surface strongly influences the pseudoperoxidase and nitrite reductase activity of electrode-bound yeast cytochrome c. The effect of hydrophobic immobilization. Bioelectrochemistry.

[58] Lancellotti L (2020). Urea-induced denaturation of immobilized yeast iso-1 cytochrome c: Role of Met80 and Tyr67 in the thermodynamics of unfolding and promotion of pseudoperoxidase and nitrite reductase activities. Electrochim. Acta.

[59] Davies DR, Plaskitt A (1971). Genetical and structural analyses of cell-wall formation in *Chlamydomonas reinhardi*. Genet. Res..

[60] Green, M. R., Sambrook, J. *Molecular Cloning: A Laboratory Manualk*. (2013).

[61] Young REB, Purton S (2014). Cytosine deaminase as a negative selectable marker for the microalgal chloroplast: A strategy for the isolation of nuclear mutations that affect chloroplast gene expression. Plant J..

[62] Kindle KL, Richards KL, Stern DB (1991). Engineering the chloroplast genome: Techniques and capabilities for chloroplast transformation in *Chlamydomonas reinhardtii*. Proc. Natl. Acad. Sci. U. S. A..

[63] Werner R, Mergenhagen D (1998). Mating type determination of *Chlamydomonas reinhardtii* by PCR. Plant Mol. Biol. Rep..

