# Supplementary material for: A PETase enzyme synthesised in the chloroplast of the microalga Chlamydomonas reinhardtii is active against post-consumer plastics
Source: Sci Rep. 2023 Jun 20;13:10028. doi: 10.1038/s41598-023-37227-5 (PMC10282039; doi:10.1038/s41598-023-37227-5)
Supplement: Supplementary file 1 — Supplementary Figures. [file 41598_2023_37227_MOESM1_ESM.docx]

**Supplementary information**

**A PETase enzyme synthesised in the chloroplast of the microalga *Chlamydomonas reinhardtii* is active against post-consumer plastics**

Giulia Di Rocco*^1^, Henry N. Taunt^2^, Marcello Berto^1^, Harry O. Jackson^2^, Daniele Piccinini^1^, Alan Carletti^1^, Giulia Scurani^3^, Niccolò Braidi^3^ and Saul Purton^2^

^1^Department of Life Sciences, University of Modena and Reggio Emilia, 41125 Modena, Italy.

^2^Algal Research Group, Department of Structural and Molecular Biology, University College London, Gower Street, London, United Kingdom

^3^Department of Chemical and Geological Sciences, University of Modena and Reggio Emilia, 41125 Modena, Italy

*Corresponding author: [giulia.dirocco@unimore.it](mailto:giulia.dirocco@unimore.it)

**
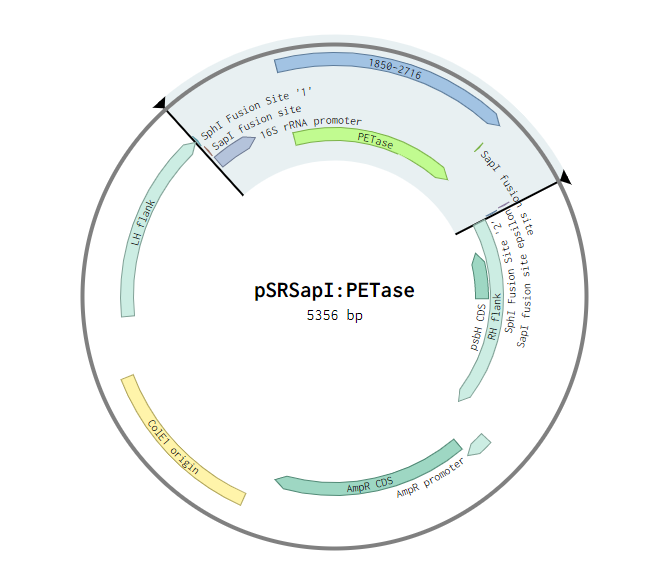
**

**Figure S1**: Transformation vector pSRSapI containing the PETase coding sequence. The entire construct is highlighted in blue and includes SapI and SphI sites added for cloning, the 16S rRNA promoter. The left and right flanks for homologous recombination and integration into the *C. reinhardtii* plastome are in green with the right flank containing *psbH* for phototrophic selection (see Figure 1A).


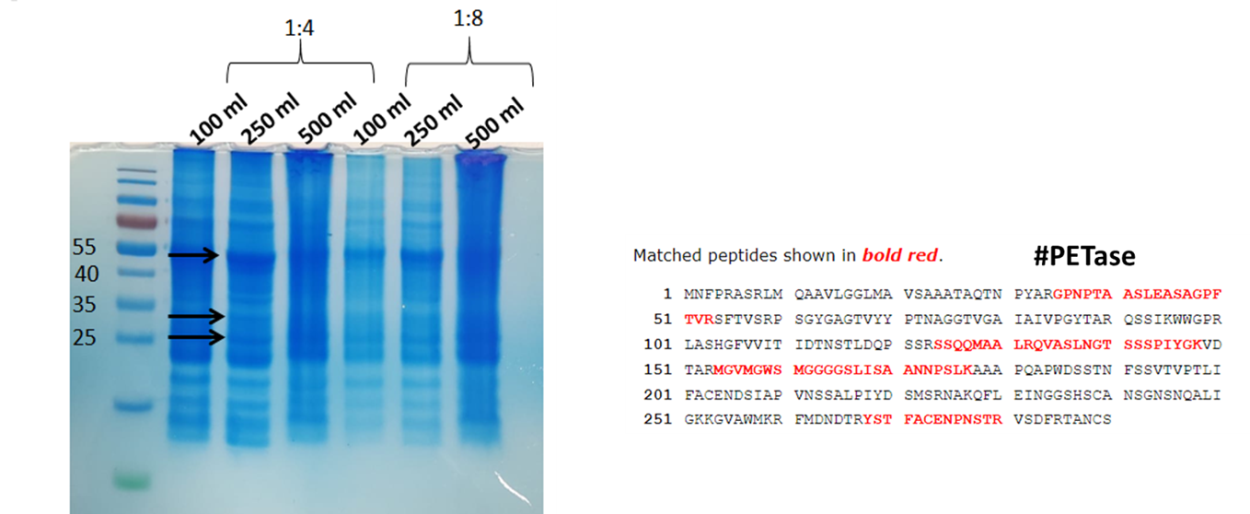


**Figure S2**: On the left, SDS polyacrylamide gel analysis of protein from algal cells grown at three scales (100mL, 250 mL and 500 mL). The arrows indicate the bands that were cut and analyzed by MS/MS. On the right sequence coverage (27%) of the complete PETase sequence from the band at ≅25kDa. The 27 residue N-terminal peptide cleaved during targeting to the thylakoid lumen is not found within the detected peptides, as predicted.


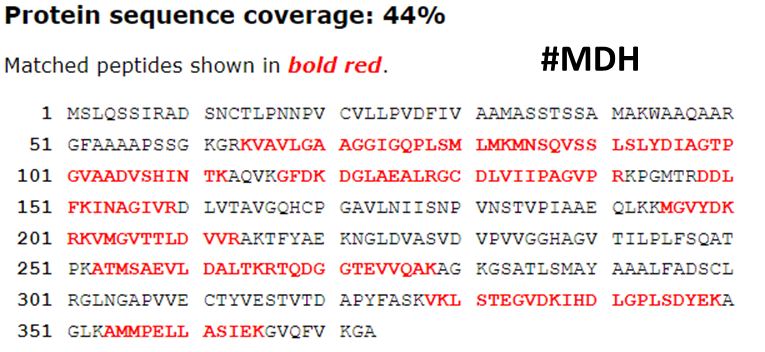


**Figure S3**: Sequence coverage (44%) for mitochondrial malate dehydrogenase precursor from *Chlamydomonas reinhardtii* (UniProtKB: locus MDHM_CHLRE, accession [Q42686](https://www.uniprot.org/uniprot/Q42686)). Cleaved transit peptide = residues 1–56.


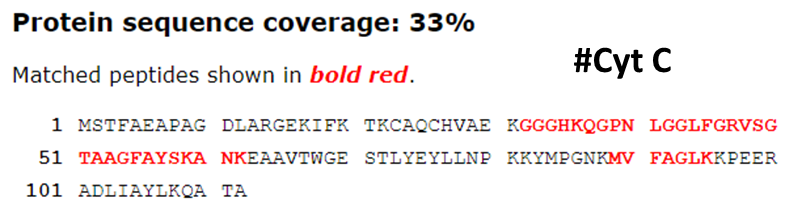


**Figure S4**: Sequence coverage (33%) of apocytochrome *c* precursor from *Chlamydomonas reinhardtii* sequence. (GenBank: [M35173](https://www.ncbi.nlm.nih.gov/nuccore/167412)).

**Table S1**. Primers used to confirm integration and homoplasmy (see Figure 1A&B).

| **Primers** |  |
| --- | --- |
| P1 (F1.long) | 5’-GTCATTGCGAAAATACTG-3’ |
| P2 (rbcL.Fn) | 5’-CGGATGTAACTCAATCGGTAG-3’ |
| P3(RY-psaR) | 5’-AACTATTTGTCTAATTTAATAACC-3’ |


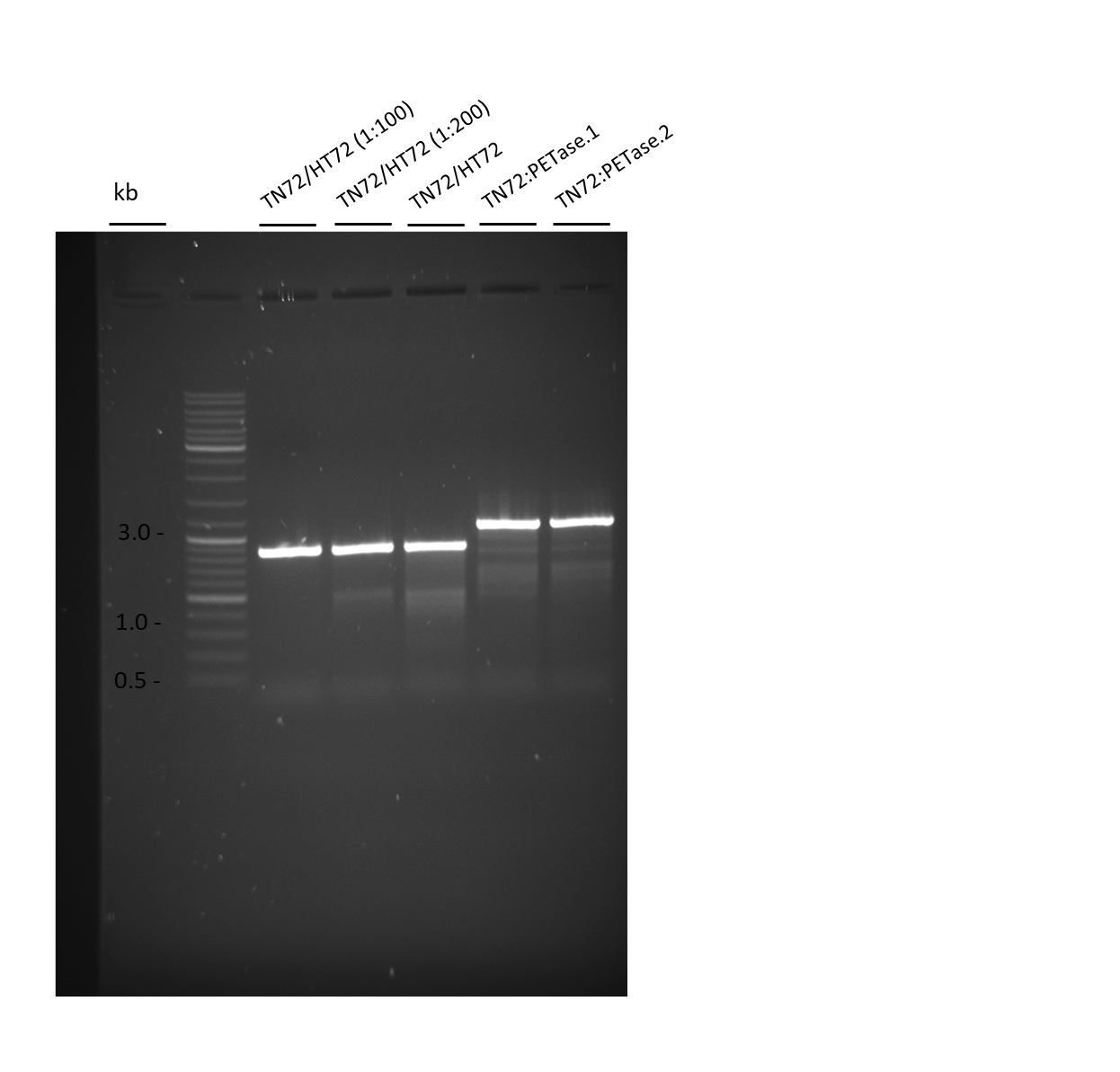


**Figure S5**: PCR results confirming the homoplasmic state of transformants TN72:PETase.1 and TN72:PETase.2. A single band of 878 bp is observed in the TN72 control, whereas a 1037 bp band is detected for the transformant lines. 1:100 and 1:200 dilutions of the TN72 DNA validate the sensitivity of the assay for detecting even a single copy of the parental DNA remaining in the transformant chloroplast. Non cropped version of the image presented in Figure 1B.


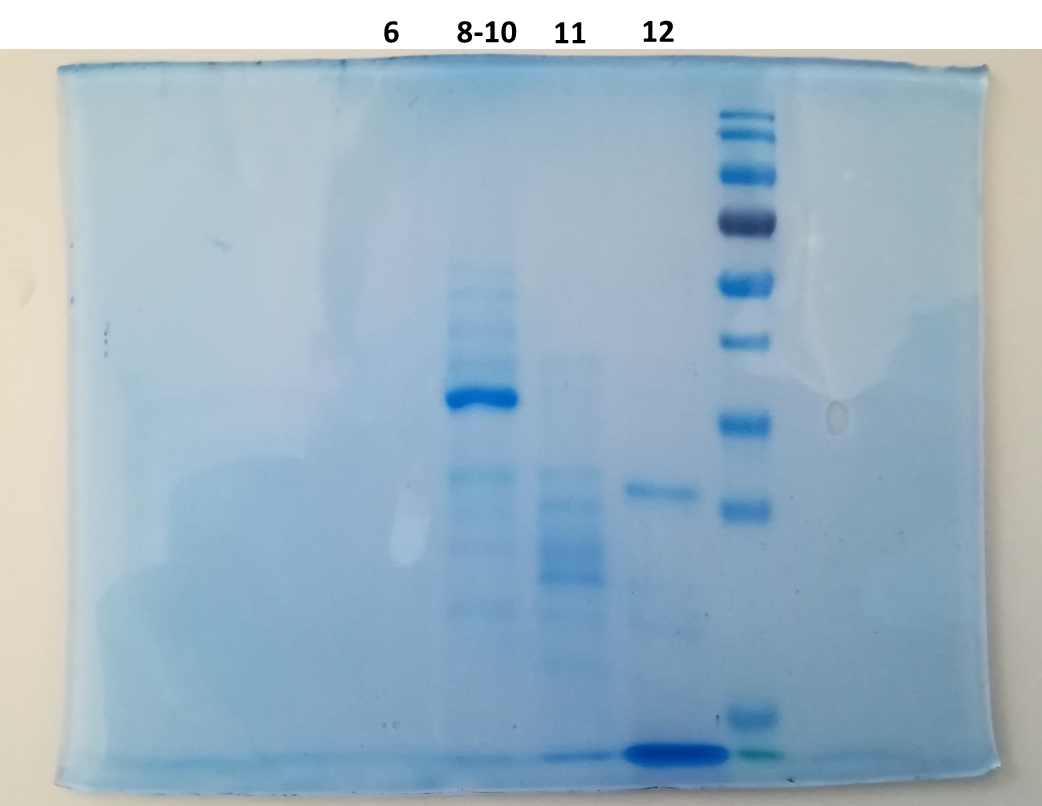


**Figure S6**: SDS-PAGE for the fractions eluted during HiLoadTM SuperdexTM 75 chromatography; the bands of MDH (malate dehydrogenase), PETase and cytochrome c were cut from the gel and the digested peptides extracted for MS analysis. Non cropped version of the image presented in Figure 2C.

**Figure S7**: Calibration curve for terephtalic acid (TPA) and for Bis(2-Hydroxyethyl) terephthalate (BHET). The calibration was obtained with a number of replicates n=3 each and with a R^2^= 0,9789 and 0,9806, respectively. From the two curves it was possible to determine the concentration of TPA and BHET released during the experiments.


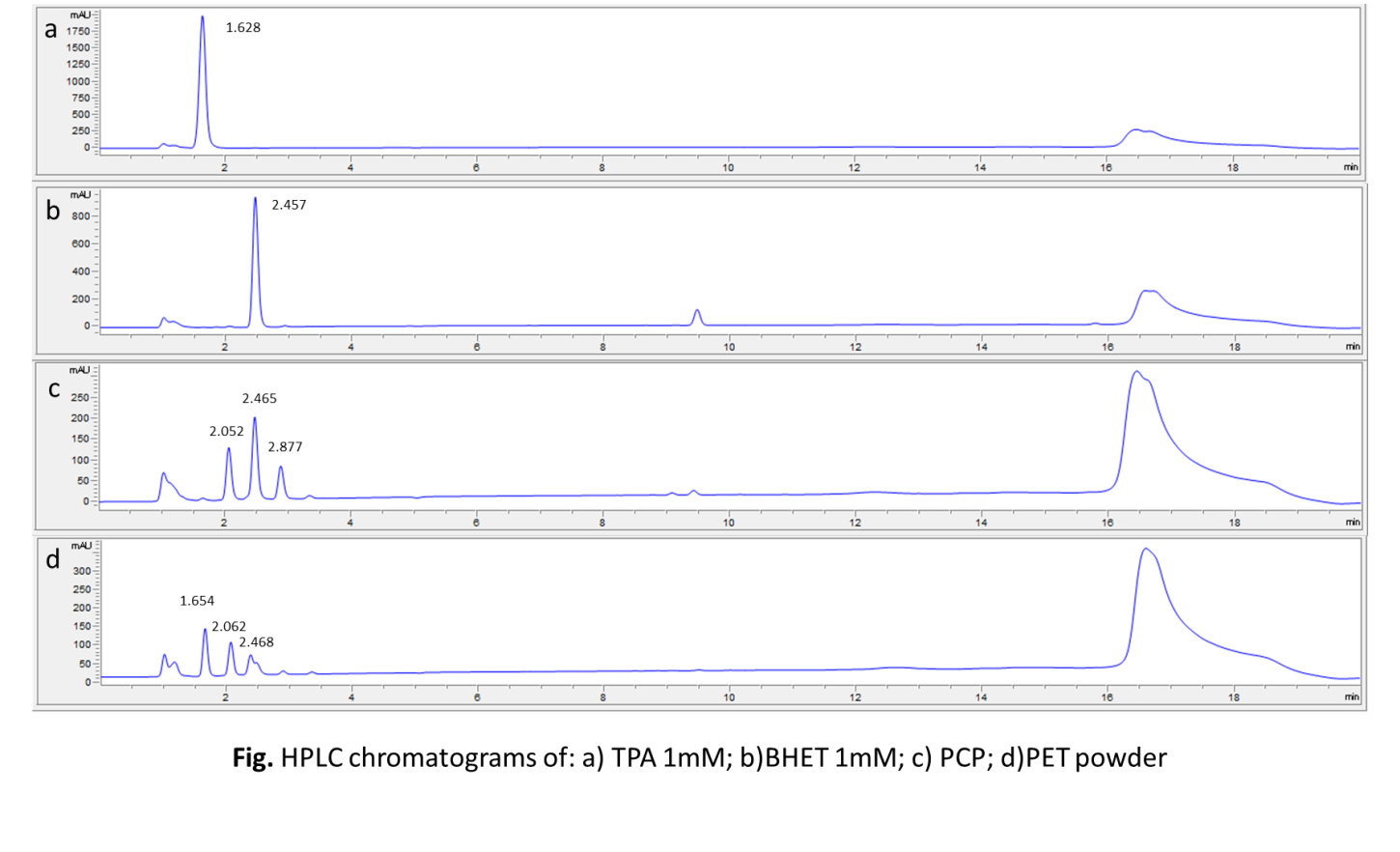


**Figure S8**: HPLC chromatograms of: a) TPA standard 1mM; b)BHET standard 1mM; c) PCP reaction supernatant; d)PET powder reaction supernatant. Highlighted in the images are the retention times for TPA t_r_=1.628; BHET t_r_=2.457; MHET t_r_=2.062


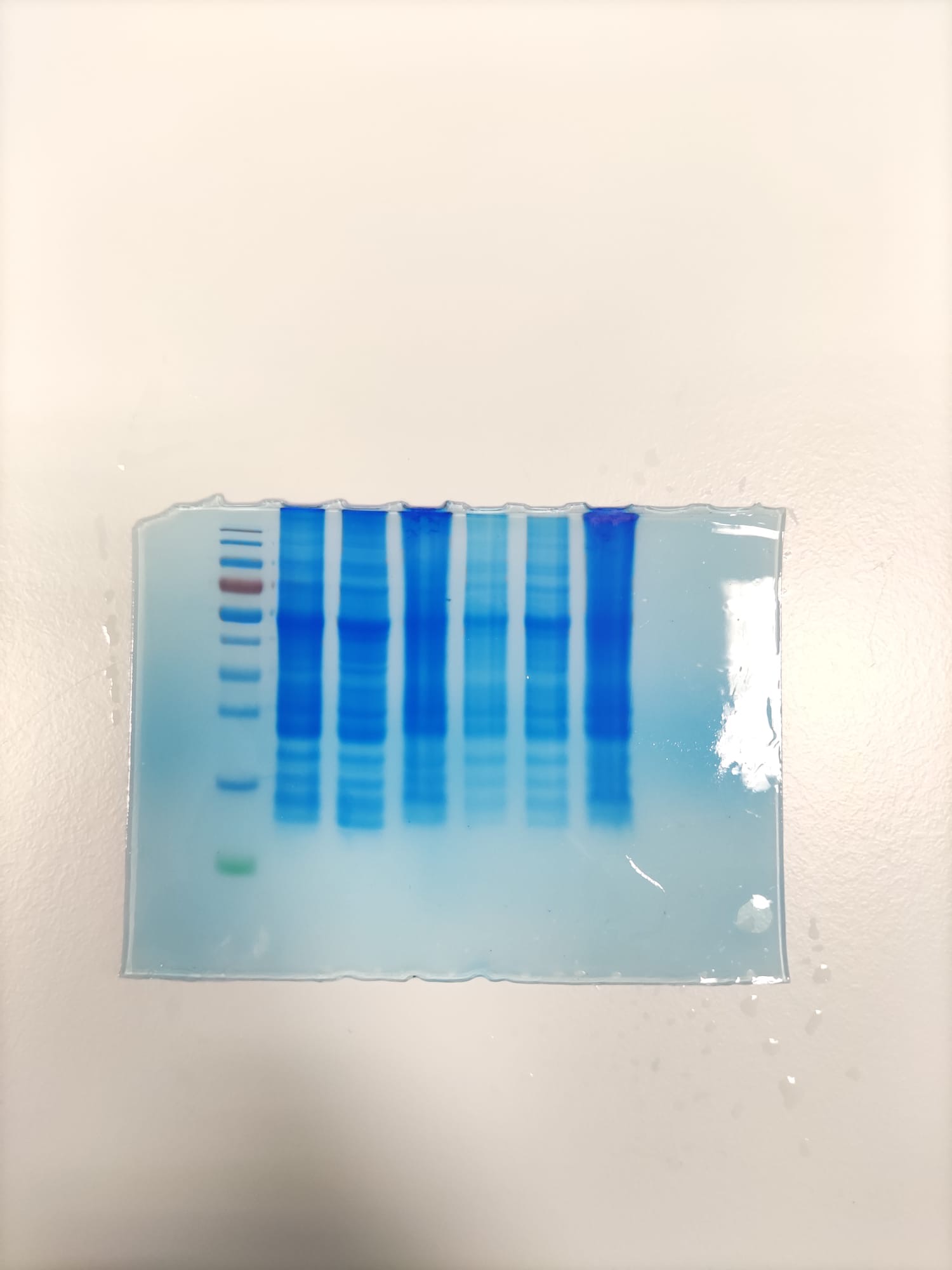


**Figure S9**: Non cropped version of the SDS polyacrylamide gel analysis of protein from algal cells grown at three scales (100mL, 250 mL and 500 mL) presented in Figure S2
